# Supplementary material for: CMImpute: cross-species and tissue imputation of species-level DNA methylation samples across mammalian species
Source: Genome Biol. 2025 May 20;26:133. doi: 10.1186/s13059-025-03561-2 (PMC12090574; doi:10.1186/s13059-025-03561-2)
Supplement: Supplementary file 5 — Additional file 5. Supplementary tables S1-S2 [file 13059_2025_3561_MOESM5_ESM.pdf]

Supplementary Tables

Cross-species and tissue imputation of species-level DNA methylation samples across mammalian species.

Emily Maciejewski, Steve Horvath, Jason Ernst

Contents

Supplementary Tables 1-2

	Pearson r All Probes	MSE All Probes	Pearson r Highest-Coverage Probes	MSE Highest-Coverage Probes
Species Baseline	68%	68%	83%	82%
Logistic Regression	78%	74%	65%	59%
Tissue Baseline	98%	98%	93%	92%
Global Baseline	97%	97%	94%	92%

Supplementary Table 1. Percentage of imputed samples where CMImpute outperforms baselines. For each baseline method (species baseline, logistic regression, tissue baseline, and global baseline top to bottom), the table displays the percentage of imputed species-tissue combination mean samples from the cross-validation analysis where CMImpute outperforms the baseline. A percentage is reported based on Pearson correlation considering all probes, MSE considering all probes, Pearson correlation restricted to the subset of highest-coverage probes, and MSE restricted to the subset of highest-coverage probes (left to right).

Hyperparameter	Option
n	8, 9, 10, 11
Layout	1, 2, 3, 4, 5
Activation Function	ReLu, Sigmoid, TanH
Learning Rate	0.001, 0.01
Epsilon	1e-7, 1e-5, 0.001, 0.1
Latent Space Dimension	2, 4, 8

Supplementary Table 2. Hyperparameters for the CVAE model. Search space for the grid search consists of all potential combinations of hyperparameter values. n is the parameter used to calculate the hidden layer dimensions and Layout corresponds to the network architecture in Additional file 1: Figure S26. Activation function used to initialize each hidden layer. Learning Rate and Epsilon are parameters in the Adam optimizer. Latent space dimension is the dimension of the latent layer.
