# Supplementary material for: CMImpute: cross-species and tissue imputation of species-level DNA methylation samples across mammalian species
Source: Genome Biol. 2025 May 20;26:133. doi: 10.1186/s13059-025-03561-2 (PMC12090574; doi:10.1186/s13059-025-03561-2)
Supplement: Supplementary file 7 — Additional file 7. Review history. The review history is available as Additional file 7 [file 13059_2025_3561_MOESM7_ESM.docx]

Review History

**First round of review**

**Reviewer 1**

**Were you able to assess all statistics in the manuscript, including the appropriateness of statistical tests used?**

**Yes.**

**Were you able to directly test the methods?**

No.

**Comments to author:**

The authors propose CMImpute, a method for imputing DNA methylation data across species. CMImpute uses a Conditional Variational Autoencoder (CVAE) model. In the training stage, CMImpute takes as input two matrices: (1) A sample-by-region matrix containing the observed methylation levels of regions targeted by probes for each sample; (2) A sample-by-label matrix containing one-hot encoded species/tissue labels for each sample. The encoder part of the CVAE transforms the input matrices into a latent space representation where a latent vector is generated for each sample. In the decoder part of the CVAE, the latent representation is used to reconstruct the input matrix by generating a sample-by-region matrix that contains the methylation levels of regions targeted by probes for each sample. In the imputation stage, a one-hot encoded label vector corresponding to a sample is passed to the trained model as input, and the model generates a vector of imputed methylation levels corresponding to all the targeted regions for that sample.

The manuscript addresses an important task. DNA methylation data is very useful in better understanding the mechanisms behind gene regulation and the imputed datasets will be very useful in getting the big picture of DNA methylation variations across tissues and species. The authors' approach is the first proposed method for cross-species and cross-tissue imputation. The manuscript is well-written and contains several important evaluations. However, there are several concerns, none of which are major.

Comments:

The authors do not compare to any of the existing approaches for imputation that were developed for single species (refs 26-33). It would be valuable to know if CMImpute is useful only in cases where few species-specific data sets are available, or if training across species improves imputation in well-profiled species. In fact, the authors essentially use CMImpute as a single-species model in several places by subtracting the species average (Figs 2f, 6d).

As we see it, the advantage of using a VAE-based model is that it produces a distribution rather than a point estimate. However, as far as I can tell the authors only used it for producing point estimates. What is the benefit of probabilistic model? Also, what is the impact of the variation introduced by sampling the latent features from a normal distribution during the imputation stage?

Page 14: The authors mentioned "The tissue and logistic regression baselines, which previously did not show species-specific signal, did show tissue-specific methylation patterns (Supplementary Fig. 18c-d,19b-c). We note that many of those patterns were not consistent with patterns seen in either the observed data or CMImpute's predictions." Comparing Fig. 6d with the supplementary Fig. 19b, the overall heatmap patterns are not drastically different between CMImpute and the Logistic Regression baseline, so the authors' statement that "..those patterns were not consistent" is not very accurate.

Following the above point, Fig. 6 is missing a heatmap corresponding to the "observed combination mean samples with species signal removed" (similar to Fig. 2d) on the full imputed dataset, so it is not possible to verify authors' claim in the above statement and see the agreement between the imputed and observed data.

Page 15, line 29-43: Are the AUC numbers mentioned in the text from Fig. 7? If yes, those numbers are a little different than what's plotted in Fig. 7.

Fig. 4a and 4b: The correlation for some probes using the species baseline model is close to perfect while that is not the case using CMImpute. And Fig. 4e and 4f show that those probes are not only in the low-variance bins, but some appear to be in the higher-variance bins. It would be nice if the authors look into those probes to see if there are some characteristics that make those probes easier to impute for the species model compared to CMImpute, or the results seen in the Fig. 4 is just an artifact of the data

Minor:
Page 13, line 17: The text actually refers to Supplementary Fig. 13b and not Fig. 5b.
Page 13, line 24: A reference to Fig. 5b is missing.

Habib Daneshpajouh, Maxwell Libbrecht
School of Computing Science, Simon Fraser University

**Reviewer 2**

**Were you able to assess all statistics in the manuscript, including the appropriateness of statistical tests used?**

Yes: I was able to assess all statistics, however I do not have enough direct training/experience in neural networks to dissect the methods used there.

**Were you able to directly test the methods?**

No.

**Comments to author:**

Major comments:

Maciejewski et al. attempts to deal with the extreme sparsity in the tissue vs. species matrix in the Mammalian Methylation Consortium, which uses a DNA methylation array consisting of 36k CpGs that are well conserved across mammals. They present a neural network machine learning model which predicts tissue/species methylation levels for individual CpGs where the specific tissue/species combination is not available. The results are compared to several naïve methods (averaging all the species across tissue or all the tissues across speciest), as well as a logistic regression model. Overall, I accept the high quality of the results as presented, since the methods used are reasonable and appear to be robust. CMImpute outperforms the competing methods (albeit sometimes by a relatively modest margin). As a one-size fits all solution, CMImpute is reasonable. However, I do have some issues regarding the interpretations, as described below. I recommend that these issues be acknowledged in the Discussion, Conclusion, and Abstract sections to put the work in context. I also suggest a few additional bioinformatics analyses that could provide additional context.

In my opinion, the focus on sample-wise correlations is somewhat obfuscating, and the real insights come from the probe-wise analyses presented in Figure 4a-f. These show that different probes behave very differently. Probes with very little tissue to tissue variability perform well in CMImpute (PCC centered around 0.8), but these probes tend to perform just as well or better under the naïve "species baseline" (Figure 4f). These include a significant fraction of the probes, if not the absolute majority. Concerningly, these probes appear less likely in the "highest-coverage" probe set, meaning they could be more susceptible to non-unique alignments (I base this on the significantly worse performance of species baseline in Figure 4a than in Figure 4b).

In contrast, probes with higher tissue-to-tissue variability perform more poorly in CMImpute (PCC centered around 0.6 in Figure 4d and 4f).

The first benchmarking figure (Figure 3) focuses on the sample-wise correlations, which seem to be driven by the probes with high species-to-species variability and low tissue-to-tissue variability. I base this conclusion on the very strong performance of "species baseline" in the Figure 3 sample-wise analyses (as well as the Figure 7 sample-wise analysis). These PCC values >0.9 would clearly justify the statement in the Abstract and Conclusion that "CMImpute achieves strong correlations". However, for readers that are interested in tissue-specific probes (which many are), a correlation of 0.6 might not be strong enough. Similarly, the Conclusion states "We demonstrate that the imputed samples maintain expected species and tissue signals useful for downstream analysis". However, I think there is a big difference in performance on species signals vs. tissue signals, and this should be clarified.

The analysis in Figure 5 and Supplemental Figure 13 focus on sample-wise correlations, but as mentioned above, these are predominantly driven by probes that are not tissue specific. It would be useful to provide similar analyses separately for the probe sets with high tissue vs. high species variability.

I suspect that the "maximum lifespan" feature is much more strongly associated with species signals and very little with tissue signals. That being the case, I think you should also show results of that analysis with the species baseline data. That would help provide the important context for this analysis.

I think that it's really important to add the context above regarding differential ability to impute species-variable vs. tissue-variable probes. This will help guide readers toward the utility and caveats of this work. I think you acknowledge the central importance of tissue-specific methylation in the opening statement of the Discussion section, when you say, "Handling of incomplete and imbalanced tissue sample coverage across the 348 species presents a significant bioinformatics challenge .. To tackle this, we introduced CMImpute".


---

Other comments:

Why do you show Figure 4g and 4h as a a scatterplot rather than showing it as a series of bar plots stratified by variance, as in Figure 4e and 4f. Wouldn't it be more intuitive and more easy to compare if they had the same visualization?

Figure 4 most labels are too tiny

The full set of imputed species-tissue combination mean samples is available only on a UCLA web server. Could you deposit this in a permanent repository such as GEO or Zenodo.

**Authors Response**

**Point-by-point responses to the reviewers’ comments:**

*Reply: We thank the reviewers for their constructive reviews. Our responses are shown below in blue.*

Reviewer #1:

The authors propose CMImpute, a method for imputing DNA methylation data across species.

CMImpute uses a Conditional Variational Autoencoder (CVAE) model. In the training stage, CMImpute takes as input two matrices: (1) A sample-by-region matrix containing the observed methylation levels of regions targeted by probes for each sample; (2) A sample-by-label matrix containing one-hot encoded species/tissue labels for each sample. The encoder part of the CVAE transforms the input matrices into a latent space representation where a latent vector is generated for each sample. In the decoder part of the CVAE, the latent representation is used to reconstruct the input matrix by generating a sample-by-region matrix that contains the methylation levels of regions targeted by probes for each sample. In the imputation stage, a one-hot encoded label vector corresponding to a sample is passed to the trained model as input, and the model generates a vector of imputed methylation levels corresponding to all the targeted regions for that sample.

The manuscript addresses an important task. DNA methylation data is very useful in better understanding the mechanisms behind gene regulation and the imputed datasets will be very useful in getting the big picture of DNA methylation variations across tissues and species. The authors' approach is the first proposed method for cross-species and cross-tissue imputation. The manuscript is well-written and contains several important evaluations. However, there are several concerns, none of which are major.

*Reply: We thank the reviewer for the summary, positive comments, and constructive feedback. We have revised the manuscript and responded to clarify our methodology and motivation and provide further context to our analyses.*

Comments:

1)	The authors do not compare to any of the existing approaches for imputation that were developed for single species (refs 26-33). It would be valuable to know if CMImpute is useful only in cases where few species-specific data sets are available, or if training across species improves imputation in well-profiled species. In fact, the authors essentially use CMImpute as a single-species model in several places by subtracting the species average (Figs 2f, 6d).

*Reply: We thank the reviewer for the comment. We did not compare to these cited methods because we do not feel they are directly applicable to the problem we were focused on of cross-species methylation imputation. We believe a comparison between CMImpute and these* *existing methods is either not applicable due to differences in a method’s outputs and overall goal or out of the scope of this study based on input data requirements, depending on the method. We go into further detail below why each existing method either does not align with the goal or is out of scope to compare to. We additionally have modified the introduction and discussion text and added a supplementary analysis to clarify the differences between CMImpute and existing methods and further highlight that CMImpute’s primary motivation is to impute methylation across the wide range of mammalian species, not just well profiled species. We will first address the components of this comment then put text modifications at the end.*

*MRM, BoostMe, DeepCpG, MRCNN, and β-VAE (1–5) (references 26 through 30 in the manuscript) impute missing or low-coverage CpG sites within existing samples as opposed to imputing a whole missing sample across all CpG sites. Thus, these methods are unable to impute methylation from an unprofiled species and tissue combination. We cite these methods as examples of machine and deep learning techniques being applied to DNA methylation imputation, but do not compare due to the different purposes and types of inputs of the methods.*

*Methods such as ChromImpute and PREDICTD (6,7) (references 31 and 33 in the manuscript), and Avocado (8) (citation added in revision), perform large-scale imputation of multiple epigenetic assays, including DNA methylation. Unlike the other methods mentioned above, which impute missing or low-coverage CpG sites, ChromImpute, PREDICTD, and Avocado are able to predict whole datasets across tissue/cell types. However, while the approach of these methods differ, both require at least one other epigenetic mark across multiple samples to allow for imputation of epigenetic marks not available in certain cell and tissue types. As most species do not have other epigenetic marks available besides DNA methylation, these existing large-scale imputation methods cannot be applied to the wide range of mammalian species profiled on the mammalian methylation array.*

*We note that we are unsure if by well-profiled species the reviewer means methylation samples covering a larger number of tissue types or if the reviewer means have various other epigenetic assays. We first address CMImpute’s utility in species with a large number of tissue types, then in species with numerous epigenetic assays other than DNA methylation.*

*With respect to species with DNA methylation samples covering a large number of tissue types profiled in the species, then the problem of not having another epigenetic assay in the tissue discussed above still exists. Even in species that are well-profiled in DNA methylation specifically with multiple tissue types available in the Mammalian Methylation Consortium dataset, there are still rarely other epigenetic marks available. Cross-species information would still be necessary to predict unseen tissue types and the existing large-scale imputation methods could not be applied. We note that we previously included an analysis regarding the impact of the amount of training data on imputation accuracy. In Figure 5, we investigated the* *sample-wise performance as a function of the number of tissue types within a target species and found that while performance increases as the number of same-tissue species increases, CMImpute still yielded high performance with limited amounts of same-species training data. This analysis demonstrates that CMImpute is able to impute unseen tissue types in both well and not well-profiled species in regards to the amount of DNA methylation profiled in that species.*

*With respect to well-profiled being in regards to the number of epigenetic assays profiled in that species, we have clarified why imputing from only DNA methylation data is still of interest even in such species. In the context of the mammalian methylation compendium if we introduced assays other than DNA methylation this could substantially confound comparisons between species as the types of epigenetic assays would vary substantially between species. Furthermore, the compendium would then represent other layers of information beyond DNA methylation. We also note in the context of the mammalian methylation array compendium that motivated this work we did not have additional epigenetic assays collected in the same samples even for what are generally considered well profiled species. Existing epigenomic methods generally assume multiple epigenetic assays are available in the same samples.*

*While imputing DNA methylation for non-well profiled species is a major goal of ours, we do believe there is also motivation for applying CMImpute to well profiled species. However, we acknowledge that it might be possible to improve predictive performance using additional information from other epigenetic assays, and now added a statement in the discussion on this point.*

*To further support our goal of imputing across all mammalian species, we added a supplementary figure analyzing CMImpute’s sample-wise performance across different phylogenetic orders. We find that CMImpute yields high performance across various phylogenetic orders, with one outlier being the order Monotremata which encompasses a very low number of imputed samples from egg-laying mammals. This analysis supports that CMImpute can effectively impute combination mean samples from a wide range of species, for which current methods are not designed. We added the text to the sample-wise results, a*

*sentence to the discussion, and the supplementary figure itself.*

*We additionally modified the text in the introduction where we introduce the previous methods to highlight their differences from CMImpute. We also modify the text to clarify that our goal is to impute methylation data in all mammalian species, not just well-profiled species or species with multiple epigenetic assays available. We copy this whole portion of the introduction later in this response.*

*In the introduction, we additionally described methCancer-gen (10) (reference 32 in the manuscript), which uses a CVAE to generate human cancer DNA methylation samples. However, this method is only able to impute samples in cancer types that are already experimentally available in some individuals and is used for data augmentation purposes. Thus, it is not able to impute DNA methylation samples in species and tissue types that have not been experimentally profiled. We cite and briefly discuss this method as an example of CVAEs being applied to DNA methylation, adding a line to further clarify the difference from CMImpute.*

*We note that we only removed the species signal by subtracting the average methylation values of same-species training samples from the full methylation values in Fig. 2d-f,6d for visualization purposes. Since the observed methylation data contains stronger species signal than tissue signal, it is difficult to qualitatively discern any tissue-specific signal from the heatmaps of observed and imputed data. By regressing out the species signal, we are able to visualize differentially methylated regions specifically correlated with tissue type. This is solely used as a visualization tool and is not considered a single-species model or used for any further downstream analyses or applications of the imputed data. We have modified the text where we discuss Fig. 2d-f to clarify this purpose.*

*Relevant text from discussion regarding the future direction of incorporating additional epigenetic assays into the model: “However, it might be possible to improve predictive performance by taking into account data from other sources, particularly for species with extensive additional epigenetic data available such as human and mouse. Future work could investigate approaches for also incorporating other biochemical data, when available, or sequence information into predictions.”*

*Relevant text from sample-wise results regarding performance across phylogenetic orders: “We additionally investigated CMImpute’s sample-wise performance across different phylogenetic orders considering all probes (Supplementary Fig. 2d) and the subset of highest-coverage probes (Supplementary Fig. 2e). Overall, CMImpute yielded high sample-wise Pearson correlations across all phylogenetic orders, with the mean correlation remaining between 0.877 and 0.935. The only outlier was the order Monotremata, which had a mean correlation of 0.806 but contained only 13 samples and encompasses two egg-laying mammals. CMImpute outperformed the species baseline across orders representing the majority of species considered and outperformed all other baselines across all phylogenetic orders (Supplementary Fig. 2d-e, Supplementary Data 1). This result demonstrates that CMImpute is able to accurately impute combination mean samples across a wide range of mammalian species.”*

*Relevant text from discussion regarding performance across phylogenetic orders: “CMImpute was able to accurately impute samples across a wide range of phylogenetic orders.”*

*Added supplementary figure (only part d and e is new from previous submission) and legend displaying performance across phylogenetic orders:*

***“Supplementary Figure 2. Sample-wise performance distributions. d-e)*** *Sample-wise Pearson correlation of imputed combination mean samples based on phylogenetic order of the imputed species for* ***d)*** *all probes and* ***e)*** *the subset of highest-coverage probes.”*

*Relevant modified text from the introduction regarding previous imputation methods. The last sentence of the first paragraph and the second paragraph up to the discussion of the coverage of a common set of CpGs has been modified from the original submitted version.*

*“The incomplete and imbalanced coverage of the experimental data thus motivates the need for computational approaches to accurately impute a DNA methylation sample representing a species and tissue type combination for which there is no experimental data available.*

*Current methods have shown that large-scale imputation of epigenetic datasets including DNA methylation (26-34) can be effective in certain contexts. For instance, some methods can impute missing or low-coverage CpG sites within existing samples, but are unable to impute a whole missing methylation sample in an unprofiled species and tissue type (26-30). Other methods can impute whole datasets for an assay when there is a different epigenetic assay* *conducted in the same sample, primarily in the context of a single species (31,33,34) or in one case in human and mouse (35). However, data from multiple epigenetic assays is not available for the vast majority of samples and species profiled by the mammalian methylation array. Furthermore, existing methods do not leverage compendia of cross-species DNA methylation data that have emerged (14,16), and in particular coverage of a common set of conserved CpGs profiled by the mammalian methylation array (14). Overall, in cases in which there is no data available in a given tissue type for a target species, such as in a less common species or tissue types that are difficult to access, methods that only consider data from a single species and thus do not harness cross-species compendia would not be able to make predictions for that tissue type.”*

*Relevant text describing how methCancer-gen differs from CMImpute: “VAEs and CVAEs have been used in various bioinformatics applications (36,37) including in the context of DNA methylation (30,32). Previous applications in the context of DNA methylation include imputing missing CpG values within an existing sample (30) and generating additional human cancer DNA methylation samples solely for data augmentation purposes when data for that cancer type is already experimentally available for some individuals (32). However, none of these existing VAE and CVAE-based approaches have been designed for or applied in the context of cross-species DNA methylation imputation and thus* *cannot impute methylation in an unprofiled* *species and tissue type.”*

*Relevant modified text regarding Fig. 2d-f: “However, when the species contribution was removed from the observed and imputed datasets by subtracting the average of all same-species training samples for visualization purposes (Fig. 2d-f, Supplementary Fig. 1c), we observed differentially methylated regions in the observed and CMImpute combination mean samples but not the species baseline-imputed samples.”*

2) As we see it, the advantage of using a VAE-based model is that it produces a distribution rather than a point estimate. However, as far as I can tell the authors only used it for producing point estimates. What is the benefit of probabilistic model?

*Reply: We thank the reviewer for their questions. In general, VAE and CVAE models are used to produce a distribution. Since the focus in this manuscript was to predict a representative sample for a species-tissue combination representing the mean methylation values within that combination, we did not use the CVAE to model a distribution across individual samples. While a non-probabilistic conditional autoencoder (CAE) could also learn the relationships between species and tissue types, we chose to use the CVAE for multiple reasons. First, CAEs are less common in the literature than CVAEs and are typically used in applications such as compression and classification (11–13), as opposed to CVAEs which are used for generation tasks in various contexts including generation of biological data (10,14–16). While we achieve* *good predictive performance with control over the species and tissue during imputation, we acknowledge that we are not currently utilizing the probabilistic model for capturing uncertainty. However, with our current model configuration and training we are not finding the uncertainty estimates to be realistically calibrated. We have added text to discuss the potential for making modifications to the model to allow for modeling uncertainty and uncertainty-aware training (17).*

*Relevant text from the discussion: “Additionally, CMImpute currently does not utilize the probabilistic latent space for tasks such as modeling uncertainty. CMImpute could be adjusted to model uncertainty in the model’s predictions due to inherent noise in the data itself, referred to as aleatoric uncertainty (41,42), presenting an interesting future direction for investigating uncertainty-aware training (43,44).”*

3) Also, what is the impact of the variation introduced by sampling the latent features from a normal distribution during the imputation stage?

*Reply: The variation introduced by sampling the latent features from a normal distribution has minimal impact on the final imputed results. In the methods subsection “Species-tissue combination mean imputation”, we explain that we chose to sample the latent features from the normal distribution so that the final imputed result is independent of any one individual sample and is instead driven by the conditional species and tissue label. We chose to sample from the standard normal distribution as the CVAE is regularized to be as close to a standard normal distribution as possible. We also verified that the specific sampled latent space values had minimal impact on the final predicted sample. To do this we used a random fold from the cross-validation analysis and imputed combination mean samples for each held-out species and tissue combination. For each species-tissue combination, we imputed 20 combination mean samples using a different random normal sampling each time. We then took the variance* *between samples of the same combination but different latent samples and samples of different combinations to show that different random normal latent sampling results in low variation between samples of the same combination (median probe variance 1.99e-05). This result aligns with the output of the CVAE being driven by the conditional label, which allows us to have control over the behavior of the final species-tissue combination mean samples. We had previously described this process in the Supplementary Fig. 27 figure legend, but have now added the description to the main text of the methods section to clarify the analysis. We provide the added text to the Methods section and the unmodified Supplementary Fig. 27 below.*

*Relevant text from the Methods section: “We investigated the impact of the specific samples from random normal on the final imputed result using a random fold from the cross-validation analysis. For each held out species-tissue combination in a fold, we imputed 20 combination mean samples using a different sample from a random normal each time. We then took the variance across combination mean samples for each probe for samples of the same combination but different latent samples and samples representing different combinations. We* *found that samples representing the same species-tissue combination but imputed using different random latent space representations had low variation (median probe variation 2e-05) across samples, while samples representing different combinations had a wider range of variation (median probe variation 0.024) accounting for the difference in species and tissue types, verifying that the specific sampled latent space values have minimal impact on the final predicted sample (Supplementary Fig. 27). Thus, as expected the imputed samples are mainly based on the overall species and tissue label inputted into the trained decoder and the final imputed combination mean samples are agnostic to individual variation.”*

***“Supplementary Figure 27. Impact of the random normal latent space sampling on the final imputation result.*** *For a random fold, each held-out combination is imputed 20 times using a different random normal sampling for the latent representation. For each species-tissue combination, the variance across the 20 samples with different random normal latent samplings was calculated. Additionally, the variance across samples from different species-tissue combinations was calculated. The boxplot shows the difference in probe variance between samples of the same combination but different latent samplings and samples of different combinations. Each box labeled with median probe variance.”*

4) Page 14: The authors mentioned "The tissue and logistic regression baselines, which previously did not show species-specific signal, did show tissue-specific methylation patterns (Supplementary Fig. 18c-d,19b-c). We note that many of those patterns were not consistent with patterns seen in either the observed data or CMImpute's predictions." Comparing Fig. 6d with the supplementary Fig. 19b, the overall heatmap patterns are not drastically different between CMImpute and the Logistic Regression baseline, so the authors' statement that "..those patterns were not consistent" is not very accurate.

*Reply: We thank the reviewer for raising this point. We recognize now that the inconsistent tissue-specific methylation patterns between the observed data and the logistic regression and tissue baselines is only clear in our quantitative AUC analysis, and thus we have removed this comment from the qualitative analysis referenced by the reviewer. While Supplementary Fig. 22d-e and 23b-c (numbers reordered with other changes in the manuscript) show that logistic regression and the tissue baseline have tissue-specific signals, the AUC analysis reveals when quantified that these tissue signals differ from the tissue-specific signals seen in the observed data. Thus, we keep the discussion of the inconsistent tissue signals between logistic regression and observed data and the tissue baseline and observed data for the AUC analysis to where we can directly quantify how the tissue and species specific signals differ.*

*Relevant text from the qualitative analysis with the sentence removed: “Unlike CMImpute, the species baseline did not capture tissue-specific methylation patterns (Supplementary Fig. 22c,23a). The tissue and logistic regression baselines, which previously did not show species-specific signal, did show tissue-specific methylation patterns (Supplementary Fig.*

*22d-e,23b-c).”*

*Relevant unmodified text from the AUC analysis: “The observed and CMImpute combination mean samples had similar tissue signals with AUC values of 0.656 and 0.667, respectively, and similar species signals with AUC values of 0.992 and 0.979, respectively. The tissue and species AUC values for combination mean samples based on logistic regression (0.750, 0.786) were higher and lower, respectively, than observed and CMImpute AUC values. As expected, the species baseline had a high species AUC value (0.993) and a low tissue AUC value (0.503), while the tissue baseline had a high tissue AUC value (0.857) and low species AUC value (0.471).”*

5)	Following the above point, Fig. 6 is missing a heatmap corresponding to the "observed combination mean samples with species signal removed" (similar to Fig. 2d) on the full imputed dataset, so it is not possible to verify authors' claim in the above statement and see the agreement between the imputed and observed data.

*Reply: We thank the reviewer for the comment. Figure 6 contains heatmaps representing the full imputed compendium of samples including the 19,786 species-tissue combinations without any observed data available. Thus, we cannot provide corresponding heatmaps for direct comparison to observed data. We instead highlight that while there is no observed data to directly compare to for these species-tissue combinations, we see similar patterns to the observed data from the cross-validation analysis with and without the species signal removed (Fig. 2a,d). These patterns include sample clustering by phylogenetic order and probe clustering by higher and lower methylated regions. We have updated the text to more clearly state that this is not a direct comparison to observed data.*

*Relevant updated text from full imputed dataset results: “As these heatmaps are based on the full imputed set of combination mean samples, there was no observed data for most samples to directly compare to. However, similar to what we previously observed when clustering based on the observed data (Fig. 2a), these heatmaps also showed sample clusters that corresponded to phylogenetic order.”*

*Relevant updated text from full imputed dataset results: “To highlight tissue-specific signal captured in observed and CMImpute-imputed values, we also clustered and visualized the methylation values and pairwise correlations based on all probes after regressing out the species contribution (Fig. 2d, Fig. 6d). This revealed clusters of samples corresponding to the same or similar tissue types and a correlated block structure corresponding to tissue types in both the observed (Supplementary Fig. 22a) and CMImpute-imputed (Supplementary Fig. 22b) combination mean samples, despite covering different sets of species-tissue combinations.”*

*We have additionally updated the supplementary figure containing the heatmaps of pairwise correlations between samples with the species signal removed to include a heatmap corresponding to the observed data. Like the original heatmaps discussed in Figure 6, we cannot make a direct comparison between the observed and imputed heatmaps as they cover different sets of species-tissue combinations. However, including the observed heatmap provides additional context to the imputed heatmaps.*

***“Supplementary Figure 22. Visualization of tissue signal in imputed samples of non-observed combinations. a)*** *Heatmap of pairwise correlations between 746 observed combination mean samples, with at least two tissue types available, with the species signal removed. White space represents undefined Pearson correlation values because of no variation in the samples.* ***b-f)*** *Heatmaps of pairwise correlations between 20,251* ***b)*** *CMImpute,* ***c)*** *species baseline,* ***d)*** *logistic regression,* ***e)*** *tissue baseline, and* ***f)*** *global baseline-imputed combination mean samples with the species signal removed (as shown in Fig. 6d). Samples are ordered based on hierarchical clustering of the difference between the full imputed samples and the species baseline samples followed by optimal leaf ordering.”*

6) Page 15, line 29-43: Are the AUC numbers mentioned in the text from Fig. 7? If yes, those numbers are a little different than what's plotted in Fig. 7.

*Reply: We thank the reviewer for bringing this to our attention. We had accidentally used an outdated version of Figure 7 in our original submission. The AUC values we referenced on page 15 were correct and our conclusions remain the same. We have replaced Figure 7 with the correct version. All other figures are up to date.*

7) Fig. 4a and 4b: The correlation for some probes using the species baseline model is close to perfect while that is not the case using CMImpute. And Fig. 4e and 4f show that those probes are not only in the low-variance bins, but some appear to be in the higher-variance bins. It would be nice if the authors look into those probes to see if there are some characteristics that make those probes easier to impute for the species model compared to CMImpute, or the results seen in the Fig. 4 is just an artifact of the data

*Reply: We thank the reviewer for this comment. The probes where the species baseline is close to perfect and thus outperforms CMImpute are probes with low tissue to tissue variability. The reason a portion of these probes with high species baseline performance are not in the lowest mean inter-tissue variance quartile bin is that a large percentage of probes have relatively low tissue to tissue variability. Thus, when the probes are split into quartiles, the first two quartiles contain probes with low mean inter-tissue variance. The probes with near perfect species baseline performance, which we consider here as probes with Pearson correlation with held-out observed data > 0.98, are restricted to these two lowest mean inter-tissue variance quartiles. The reason these probes span across all inter-combination and mean inter*-species variance quartiles is that probes with high variance across combinations and species do not necessarily have high variance across tissue types, meaning a subset of higher inter-combination or mean inter-species variance probes could have lower mean inter-tissue variance and near perfect species baseline performance.

*To demonstrate these conclusions, we have added two supplementary figures. The first figure we discuss contains histograms representing the distribution of probe-wise variances for each variance metric and red vertical lines delineating the location of probe-wise variance quartiles. These plots demonstrate for the mean inter-tissue variance metric (a,d), which is the variance metric most associated with the species baseline performance, the first two quartiles contain probes with lower inter-tissue variance within a species (i.e. mean inter-tissue variance < 0.005), thus explaining how the species baseline could yield near perfect performance in not just the lowest quartile. For the inter-combination and mean inter-species variance (b-c, e-f), the quartiles are more spread out with the first through third quartiles spanning a similar range of variances.*

*Added supplementary figure and figure legend:*

***“Supplementary Figure 5. Probe-wise variance distributions with quartiles. a-f)*** *Histogram showing the distribution of probe-wise variances in the observed data. Each histogram has five red vertical lines denoting the boundaries of the quartiles of that variance metric.* ***a-c)*** *Distributions showing the* ***a)*** *mean inter-tissue,* ***b)*** *inter-combination, and* ***c)*** *mean inter-species variances across the subset of highest-coverage probes.* ***d-f)*** *Corresponding variance distribution plots for all probes.”*

*Modified text referencing the added supplementary figure: “To analyze probe-wise performance as a function of probe variance, we used variance quartiles for each type of variance where each quartile contains one fourth of the probes being considered (Supplementary Fig. 5).”*

*With this context of the distribution of the probe-wise variance bins, we further investigated the species* *baseline performance across bins from Fig. 4e, Supplementary Fig. 6b, and Supplementary Fig. 7b (probe-wise Pearson correlation boxplots for mean inter-tissue, inter-combination, and mean inter-species variance quartiles, respectively). For the mean inter-tissue variance metric (Fig. 4e), these probes with near perfect species baseline performance are in the first and second quartiles with a maximum species baseline performance within the variance bins of 0.995 and 0.981, respectively. The third and fourth inter-tissue variance quartiles have no near perfect probes, with the maximum species baseline*

*performance within these two variance quartiles being 0.931 and 0.856, respectively. Moreover, CMImpute outperforms the species baseline for a large majority of probes in these higher variance quartiles, with a higher performance for 78% of the probes in the third quartile and 98% of probes in the fourth quartile. This aligns with the probe-wise variance distributions discussed above and the conclusion that the species baseline can yield high performance on probes with minimal variation between tissue types within a species, but perform worse as the tissue variance increases.*

*However, as the reviewer pointed out, there were probes in each inter-combination (Supplementary Fig. 6b) and mean inter-species (Supplementary Fig. 7b) quartile, including higher variance quartiles, where the species baseline had near perfect performance. The maximum species baseline performance within each variance quartile remained greater than 0.988 for both the inter-combination and mean inter-species variance metrics, demonstrating that there was at least one probe with near perfect correlation in each quartile. However, probes with higher inter-combination or mean inter-species variance do not necessarily have high variance among tissue types within a species. To demonstrate that the species baseline only achieves high performance or outperforms CMImpute on probes with low mean inter-tissue variance, we have added a supplementary analysis investigating probes where CMImpute outperforms the species baseline and vice versa, similar to the sample-wise analysis in Supplementary Fig. 3. We investigated the probe variance distributions for each variance metric among the subset of probes where the species baseline outperforms CMImpute, which encompasses the subset of probes where the species baseline performance is near perfect, and vice versa. This analysis further demonstrates that the species baseline only outperforms CMImpute on probes with low mean inter-tissue variance. Some of these probes also have low inter-combination or mean inter-species variance, however there is a wider range of inter-combination and mean inter-species variances among this probe subset. This analysis along with the overall probe variance distributions explains why there are probes with high species baseline performance not solely in the lowest variance bins.*

*Relevant added text related to this analysis: “We also investigated specific probes where CMImpute outperformed the species baseline and vice versa, and similarly for logistic regression, across all probes and the subset of highest-coverage probes. Probes where CMImpute outperformed the species baseline had higher mean inter-tissue variance (Supplementary Fig. 15a,c) and probes where CMImpute* *outperformed logistic regression had lower mean inter-tissue variance (Supplementary Fig. 15b,d). These results demonstrate CMImpute generally outperformed baselines except for the case of the species baseline for probes with low tissue to tissue variability and that overall probe-wise performance is associated with probe variance between different tissue types.” Added supplementary figure and figure legend:*

***“Supplementary Figure 15. Comparison of subset of probes where species baseline and logistic regression outperform CMImpute and vice versa. a-d)*** *The probe-wise variance of probes in the* subset *of highest-coverage (left) and overall probes (right) for mean inter-tissue, inter-combination, and mean inter-species variance in the subsets of probes where* ***a)*** *CMImpute yields a higher probe-wise correlation than the species baseline,* ***b)*** *CMImpute yields a higher probe-wise correlation than logistic regression,* ***c)*** *CMImpute yields a lower MSE than the species baseline, and* ***d)*** *CMImpute yields a lower MSE than logistic regression and vice versa.”*

Minor:

Page 13, line 17: The text actually refers to Supplementary Fig. 13b and not Fig. 5b.

Page 13, line 24: A reference to Fig. 5b is missing.

*Reply: We thank the reviewer for bringing this to our attention. We have updated the figure references where the reviewer noted to refer to Fig. 5b when discussing solely CMImpute’s performance (page 13 line 24 before revisions) and Supplementary Fig. 17b (previously 13b) when discussing CMImpute’s performance relative to the baselines (page 13 line 17 before revisions).*

Habib Daneshpajouh, Maxwell Libbrecht

School of Computing Science, Simon Fraser University

*Reply: We thank the reviewer again for their helpful questions and comments. As described above we made revisions to the manuscript text, added supplementary figures, and adjusted existing figures in response to the reviewer comments. We believe these updates improved the manuscript and provide further clarification and explanation to our results.*

Reviewer #2:

Major comments:

Maciejewski et al. attempts to deal with the extreme sparsity in the tissue vs. species matrix in the Mammalian Methylation Consortium, which uses a DNA methylation array consisting of 36k CpGs that are well conserved across mammals. They present a neural network machine learning model which predicts tissue/species methylation levels for individual CpGs where the specific tissue/species combination is not available. The results are compared to several naïve methods (averaging all the species across tissue or all the tissues across species), as well as a logistic regression model. Overall, I accept the high quality of the results as presented, since the methods used are reasonable and appear to be robust. CMImpute outperforms the competing methods (albeit sometimes by a relatively modest margin). As a one-size fits all solution, CMImpute is reasonable. However, I do have some issues regarding the interpretations, as described below. I recommend that these issues be acknowledged in the Discussion, Conclusion, and Abstract sections to put the work in context. I also suggest a few additional bioinformatics analyses that could provide additional context.

*Reply: We thank the reviewer for their positive comments regarding our approach. We also thank the reviewer for the constructive comments to which we respond to below regarding acknowledgement and explanation of probe-wise performance and the addition of bioinformatic analyses.*

In my opinion, the focus on sample-wise correlations is somewhat obfuscating, and the real insights come from the probe-wise analyses presented in Figure 4a-f. These show that different probes behave very differently. Probes with very little tissue to tissue variability perform well in CMImpute (PCC centered around 0.8), but these probes tend to perform just as well or better under the naïve "species baseline" (Figure 4f). These include a significant fraction of the probes, if not the absolute majority.

*Reply: We thank the reviewer for the comment. We agree that while CMImpute yields high performance on probes with low tissue to tissue variability, the species baseline also yields high performance on this subset. We have added supplementary figures and modified our discussion of the probe-wise performance results to further elaborate on CMImpute’s performance across different probes, particularly in reference to the species baseline.*

*As the reviewer mentions, this subset of probes with low tissue variability makes up a significant fraction of the total probes. To make this clearer and to provide additional context to our analysis, we have added a supplementary figure with the distribution of probe-wise variances for each variance metric and red vertical lines delineating the location of the probe-wise variance quartiles. This figure illustrates that a large portion of probes have low mean inter-tissue variance, particularly for the first two variance quartiles, and provides a visualization of the range and distributions of variance values being discussed when comparing probe-wise performance across different variance quartiles represented in Fig. 4e and our other boxplots.*

*Added text referencing variance distribution figure: “To analyze probe-wise performance as a function of probe variance, we used variance quartiles for each type of variance where each quartile contains one fourth of the probes being considered (Supplementary Fig. 5).” Added supplementary figure and figure legend:*

***“Supplementary Figure 5. Probe-wise variance distributions with quartiles. a-f)*** *Histogram showing the distribution of probe-wise variances in the observed data. Each histogram has five red vertical lines denoting the boundaries of the quartiles of that variance metric.* ***a-c)*** *Distributions showing the* ***a)*** *mean inter-tissue,* ***b)*** *inter-combination, and* ***c)*** *mean inter-species variances across the subset of highest-coverage probes.* ***d-f)*** *Corresponding variance distribution plots for all probes.”*

*We additionally modified the text to further elucidate that while CMImpute does perform well on probes with low tissue to tissue variability, the species baseline performs as well if not better. The modified text makes it clearer that CMImpute only consistently outperforms the species baseline in the top two variance quartiles. This text references Supplementary Fig. 5 to further show that the first two mean inter-tissue variance quartiles represent probes with low tissue to tissue variability. We additionally discussed the species baseline performance on low mean inter-tissue variance probes in the mean squared error analysis by stating that the species baseline more accurately imputed probes that contain limited tissue-specific activity.*

*Relevant modified text: “For mean inter-tissue variance considering the subset of highest-coverage probes, CMImpute consistently had a higher mean correlation and lower mean MSE than the species baseline in all but the lowest variance quartile (Fig. 4c,e, Supplementary Fig. 5a). CMImpute also had a higher median correlation in the top two variance quartiles. While the species baseline relative to* *CMImpute more accurately imputed probes with low mean inter-tissue variance, it less accurately imputed probes of higher mean inter-tissue variance, which contain greater tissue-specific activity signal.”*

*Finally, we have added a supplementary analysis that investigates the probe-wise variance of probes where CMImpute outperforms the species baseline and vice versa. This analysis further supports the conclusion that the species baseline only outperforms CMImpute on probes with low tissue to tissue variability. We additionally discuss the set of probes where CMImpute outperforms logistic regression and vice versa as logistic regression is the next highest performing baseline.*

*Relevant added text related to this analysis: “We also investigated specific probes where CMImpute outperformed the species baseline and vice versa, and similarly for logistic regression, across all probes and the subset of highest-coverage probes. Probes where CMImpute outperformed the species baseline had higher mean inter-tissue variance (Supplementary Fig. 15a,c) and probes where CMImpute outperformed logistic regression had lower mean inter-tissue variance (Supplementary Fig. 15b,d). These results demonstrate CMImpute generally outperformed baselines except for the case of the species baseline for probes with low tissue to tissue variability and that overall probe-wise performance is associated with probe variance between different tissue types.”*

*We included this figure in the response to reviewer 1 comment 6 but provide again in this response for continuity.*

*Added supplementary figure and figure legend:*

***“Supplementary Figure 15. Comparison of subset of probes where species baseline and logistic regression outperform CMImpute and vice versa. a-d)*** *The probe-wise variance of probes in the subset of highest-coverage (left) and overall probes (right) for mean inter-tissue, inter-combination, and mean inter-species variance in the subsets of probes where* ***a)*** *CMImpute yields a higher probe-wise correlation than the species baseline,* ***b)*** *CMImpute yields a higher probe-wise correlation than logistic regression,* ***c)*** *CMImpute yields a lower MSE than the species baseline, and* ***d)*** *CMImpute yields a lower MSE than logistic regression and vice versa.”*

Concerningly, these probes appear less likely in the "highest-coverage" probe set, meaning they could be more susceptible to non-unique alignments (I base this on the significantly worse performance of species baseline in Figure 4a than in Figure 4b).

*Reply: We thank the reviewer for their comment. Probes with lower tissue to tissue variability are less likely to be in the “highest-coverage” probe set, however this is expected and we do not consider it a concern. Probes that are mappable in a certain species have a greater likelihood of capturing methylation signal related to characteristics within the species such as differences between tissue types. On the other hand, probes that are not mappable in a species, either due to sequence divergence or a non-unique alignment, are unable to measure signals related to a specific genomic position and thus are unlikely to capture differential tissue signals. The subset of highest-coverage probes consists of probes that are mappable in a substantial number of species and thus the variability of these probes across the species and tissue combinations is more likely to account for characteristics present within species as opposed to just species mappability differences. Thus, even though a fraction of the probes in the subset of highest-coverage probes still have low tissue to tissue variability as some probes are not located in a differentially methylated tissue region, the highest-coverage probe set is more likely to have probes with higher tissue to tissue variability.*

*We explained why we prioritize the subset of highest-coverage probes for the probe-wise performance analysis.*

*Relevant text from the probe-wise performance results: “Our primary evaluation was for the subset of highest-coverage probes since for these probes, methylation values across samples would less likely be driven by differences in mappability across species.”*

*Additionally, we added a sentence to the methods section where we define the probe subset to acknowledge that if a probe is not mappable in a species, it is less likely to capture tissue-specific signals.*

*Relevant added text from the methods section: “If a probe does not work in a species, it is also not expected to reflect within species methylation signals, such as differential tissue signals.”*

In contrast, probes with higher tissue-to-tissue variability perform more poorly in CMImpute (PCC centered around 0.6 in Figure 4d and 4f).

*Reply: We thank the reviewer for raising this point. We respond to this comment along with the next comment below.*

The first benchmarking figure (Figure 3) focuses on the sample-wise correlations, which seem to be driven by the probes with high species-to-species variability and low tissue-to-tissue variability. I base this conclusion on the very strong performance of "species baseline" in the Figure 3 sample-wise analyses (as well as the Figure 7 sample-wise analysis). These PCC values >0.9 would clearly justify the statement in the Abstract and Conclusion that "CMImpute achieves strong correlations". However, for readers that are interested in tissue-specific probes (which many are), a correlation of 0.6 might not be strong enough. Similarly, the Conclusion states "We demonstrate that the imputed samples maintain expected species and tissue signals useful for downstream analysis". However, I think there is a big difference in performance on species signals vs. tissue signals, and this should be clarified.

*Reply: We thank the reviewer for their suggestion. We have added text throughout the Results, Discussion, and Conclusion to put the probe-wise performance, particularly in regards to the tissue signal and tissue to tissue variability, in greater context. We previously exclusively focused on comparing CMImpute’s probe-wise performance to the baseline’s performance and did not discuss the difference in Pearson correlation values between the sample-wise and probe-wise correlation analyses. While we still keep this discussion of benchmarking CMImpute’s performance relative to the baselines, particularly noting that CMImpute outperforms the species baseline on tissue-specific probes, we have now added text to discuss CMImpute’s overall performance across different types of probes without respect to the baselines. In particular, we acknowledge that probewise-performance can decrease on probes with higher tissue-to-tissue variability. We additionally have added text acknowledging the difference in CMImpute’s correlation values between the sample-wise and probe-wise correlations.*

*In describing the probe-wise performance results, we have added text acknowledging the difference between the sample-wise and probe-wise correlations of approximately 0.9 and 0.6, respectively. We specifically acknowledge that the sample-wise correlations are higher than the probe-wise correlations. We note that this could partially be due to Pearson correlation being less informative on lower variance probes. For the MSE we observed similar values for sample and probe-wise performance: “While CMImpute significantly outperformed the baselines (Fig. 4a,b), as we would expect we observed lower absolute correlations of 0.623 for the subset of highest-coverage probes and 0.688 for all probes than the sample-wise correlation of 0.932 for the subset of highest-coverage probes and 0.920 for all probes (Fig. 3a, Supplementary Fig. 2a). We note that for probes that have almost no variance across combination mean samples, we would expect the probe-wise Pearson correlation to be less informative and have low correlation values. We additionally evaluated probe-wise performance using MSE and found that CMImpute significantly outperformed the baselines for both all probes and the subset of highest-coverage probes with mean MSEs of 0.0171 and 0.0148, respectively (Supplementary Fig. 4). Unlike CMImpute’s probe-wise correlation, these mean MSE values are equal to the mean sample-wise MSE* *values (Supplementary Fig. 2b-c).”*

*In addition to discussing the difference in absolute probe-wise and sample-wise performance, we have added text providing additional explanation of CMImpute’s performance across different probe variances instead of primarily discussing CMImpute’s performance relative to the baselines.*

*When discussing the probe-wise Pearson correlation across mean inter-tissue variance quartiles, we have modified the text to acknowledge that while we outperform the species baseline on higher varying probes, the performance does decrease as mean inter-tissue variance increases: “For mean inter-tissue* *variance considering the subset of highest-coverage probes, CMImpute consistently had a higher mean correlation and lower mean MSE than the species baseline in all but the lowest variance quartile (Fig. 4c,e, Supplementary Fig. 5a). CMImpute also had a higher median correlation in the top two variance quartiles. While the species baseline relative to CMImpute more accurately imputed probes with low mean inter-tissue variance, it less accurately imputed probes of higher mean inter-tissue variance, which contain greater tissue-specific activity signal. We also note that while CMImpute outperformed the species baseline on these higher varying probes, CMImpute’s performance did decrease as the mean inter-tissue probe variance increases, with the mean correlation going from 0.692 to 0.589 and the mean MSE going from 0.005 to 0.032 in the lowest and highest variance quartiles, respectively.”*

*While the reviewer comment pertains to probes that vary across tissue types, we have additionally modified the discussion of the probe-wise Pearson correlation across inter-combination and mean inter-species variance quartiles. We have added specific performance values instead of solely stating that we outperform the baselines: “This revealed that for both these variance types, CMImpute consistently had a higher mean correlation than the baselines across all variance quartiles with a minimum mean correlation of 0.571 in the lowest variance quartile and maximum mean correlation of 0.667 in the highest variance* *quartile across both variance measures compared to the species baseline mean correlations of 0.558 and 0.550, respectively (Supplementary Fig. 6a-b,7a-b,8a-b). All other baselines yielded lower performance than the species baseline. These results were also consistent with those from the probe-wise MSE metric, where CMImpute had a lower mean MSE for all inter-combination and mean inter-species variance quartiles (Supplementary Fig. 9,10a-b).”*

*In the discussion, we modify the text to delineate the discussion of sample-wise and probe-wise performance. We additionally add text, summarizing the new text from the probe-wise results section, acknowledging that the probe-wise performance does decrease as mean inter-tissue variance increases: “CMImpute yielded reasonable sample-wise performance that was better than the baselines when limited same-species and same-tissue information for the target species-tissue combination was available, and as expected performance increased with additional same-species or same-tissue information available. CMImpute’s probe-wise performance was robust to lower and higher variance probes compared to the baselines.*

*However, despite the relative robustness we note that as tissue to tissue variability increased,*

*CMImpute’s probe-wise performance did decrease.”*

*In the conclusion, we modify the text to discuss the sample-wise and probe-wise correlations separately and clarify that the strong probewise-performance is relative to the baselines: “We have shown that CMImpute achieves a strong sample-wise correlation and low MSE with observed values. We have also shown it surpasses several baseline methods for those measures and for probe-wise correlation and MSE.”*

*In the abstract, we modified the text to only use “strong correlation” to refer to the sample-wise performance: “We demonstrate that CMImpute achieves strong sample-wise correlation between imputed and observed values”.*

The analysis in Figure 5 and Supplemental Figure 13 focus on sample-wise correlations, but as mentioned above, these are predominantly driven by probes that are not tissue specific. It would be useful to provide similar analyses separately for the probe sets with high tissue vs. high species variability.

*Reply: We thank the reviewer for their suggestion. To demonstrate that the conclusions we draw regarding the impact of the amount of same-species and same-tissue training data can also be seen with tissue specific probes, we performed similar analyses to Figure 5 and Supplementary Figure 17 (previously 13) using different probe subsets. Since the amount of training data is a sample-specific characteristic, we performed the sample-wise correlation analysis of CMImpute and baseline performance when considering different amounts of available same-species and same-tissue data, but now considering the subset of higher variance probes for each of the three variance metrics we are considering in this study. For each variance metric, we defined a corresponding set of higher variance probes as the 18,746 probes with variance greater than the median variance for that metric. We took the sample-wise correlations between the imputed and observed values across these subsets of probes. For each variance metric as a function of the number of tissue types available in the target species and the number of species available in the target tissue during training, CMImpute outperforms the baselines demonstrating that the original conclusion from Figure 5 and Supplementary Figure 17, which was based on all probes thus encompassing both* *higher and lower variance, can also be drawn when using exclusively probes with higher variance across species and tissue types. We note that while logistic regression does slightly better (less than 0.01 correlation) than CMImpute on higher variance probes when considering the maximum number of tissue types available within a target species in our cross-validation study, CMImpute outperforms logistic regression on all other amounts of training data. This supports that CMImpute is more robust to lower amounts of same-species training data than logistic regression. We have modified the results section to include the analyses on higher variance probes and added a supplementary figure.*

*Relevant modified text from results on sample-wise performance as a function of the number of same-species tissues: “This result, which was also seen when considering the sample-wise correlation across only higher variance probes (Supplementary Fig. 16a-c), was consistent with CMImpute’s improved performance with additional tissue types being driven by the additional available training data and not differences in the observed variability across individuals within the species and tissue combinations. Consistent with results based on all imputed combination mean samples (Fig. 3a), CMImpute also generally outperformed the baselines for subsets of imputed samples as a function of the amount of available training data across all probes (Supplementary Fig. 17a). CMImpute also outperformed baselines when restricted to higher variance probes, particularly on combination mean samples with less same-species tissues, which make up the majority of samples in the cross-validation analysis (Supplementary Fig.16a-c).”*

*Relevant modified text from results on sample-wise performance as a function of the number of same-tissue species: “This trend was also seen when considering the sample-wise correlation across only higher variance probes (Supplementary Fig. 16d-f). Consistent with the results from the evaluation as a function of the number of tissue types in the target species, CMImpute outperformed the baselines in sample-wise imputation performance evaluations as a function of the number of species for the target tissue type across all probes (Supplementary Fig. 17b). This was not only due to CMImpute’s performance on lower variance probes as it also outperformed the baselines when restricting to higher variance probes (Supplementary Fig. 16d-f).”*

*“****Supplementary Fig. 16. Impact of the amount of available training data on CMImpute and baseline performance across higher variance probes. a-c)*** *Sample-wise Pearson correlation across a subset of* ***a)*** *18,746 probes with a mean inter-tissue variance > 0.004 (median mean inter-tissue variance),* ***b)*** *18,746 probes with an inter-combination variance > 0.031 (median inter-combination variance), and* ***c)*** *18,746 probes with a mean inter-species variance > 0.022 (median mean inter-species variance) based on the number of tissue types available in the target species during training. Same format as Supplementary Fig. 17a.* ***d-f)*** *Sample-wise Pearson correlation across the corresponding set of higher variance probes in a-c based on the number of species available during training in the target tissue. Same format as Supplementary Fig. 17b.”*

I suspect that the "maximum lifespan" feature is much more strongly associated with species signals and very little with tissue signals. That being the case, I think you should also show results of that analysis with the species baseline data. That would help provide the important context for this analysis.

*Reply: We thank the reviewer for their suggestion. We have repeated the maximum lifespan prediction in both the tissue-agnostic and tissue-specific settings using the species baseline-imputed data to provide context to the CMImpute results. As the maximum lifespan is a species-level characteristic and thus is more strongly associated with species signals, the observed, CMImpute-imputed, and species baseline-imputed data all yield similar performance based on Pearson correlation and mean squared error between the predicted and reported log-maximum lifespan values. However, we note that in the tissue-specific setting the species baseline predicts the same maximum lifespan value for each tissue type within a species. This lack of tissue specificity makes it impossible to perform a tissue-level analysis regarding a species’ maximum lifespan or to perform any other downstream predictions regarding tissue types, which restricts the utility of the species baseline’s predictions to exclusively species-level downstream analyses. We have updated the results section and supplementary figures to include the species baseline analysis.*

*Relevant text from the maximum lifespan results: “As the maximum lifespan is a species-level characteristic, we additionally performed these evaluations using the species baseline’s imputed data. The species baseline achieved similar performance to the observed and CMImpute-imputed data in the tissue-agnostic (correlation 0.831, median MSE 0.064, Supplementary Fig. 25a,c-d) and tissue-specific (correlation 0.817, median MSE 0.063, Supplementary Fig. 25b) settings. However, the species baseline predicts the same maximum lifespan for each tissue type within a species removing the possibility of performing a tissue-level analysis regarding species’ maximum lifespan or any other downstream analysis regarding tissue type. In general, this lack of tissue specificity may restrict the utility of the species baseline’s predictions.”*

*Updated supplementary figure and figure legend:*

***“Supplementary Figure 25. Prediction of species’ maximum lifespan using combination mean samples from different tissue types. a)*** *Boxpot comparing the log-maximum lifespan prediction MSE using observed CMImpute-imputed, and species baseline-imputed averages of combination mean samples within each species. Both the imputed and experimentally-profiled combination mean samples span the same 114 species.* ***b)*** *Same as a but comparing the log-maximum lifespan prediction mean squared error using observed, CMImpute, and species baseline species-tissue combination mean samples. The 441 observed combination mean samples span all observed tissues and the 6,285 CMImpute and species baseline-imputed combination mean samples span all non-observed tissues across the 114 species.* ***c)*** *Leave-one-species-out linear regression analysis using species-average samples similar to Fig. 8b. Average methylation calculated over species baseline-imputed species-tissue combination mean samples. Predicted log-maximum lifespan (x-axis) plotted against the reported log-maximum lifespan (y-axis).* ***d)*** *Comparison of maximum lifespan predictions based on average species methylation samples between using observed and species baseline-imputed data similar to Fig. 8c.”*

I think that it's really important to add the context above regarding differential ability to impute species-variable vs. tissue-variable probes. This will help guide readers toward the utility and caveats of this work. I think you acknowledge the central importance of tissue-specific methylation in the opening statement of the Discussion section, when you say, "Handling of incomplete and imbalanced tissue sample coverage across the 348 species presents a significant bioinformatics challenge .. To tackle this, we introduced CMImpute".

*Reply: We thank the reviewer again for their helpful comments and suggestions. As described above we made a number of revisions to the manuscript text and added supplementary figures and analyses to address the reviewer’s concerns. We believe these updates improved the manuscript and provided context to our results.*

---

Other comments:

1)	Why do you show Figure 4g and 4h as a a scatterplot rather than showing it as a series of bar plots stratified by variance, as in Figure 4e and 4f. Wouldn't it be more intuitive and more easy to compare if they had the same visualization?

Figure 4 most labels are too tiny

*Reply: We thank the reviewer for the comment. We have updated Figure 4 to have more consistency between the probe-wise Pearson correlation and mean squared error figures by adding box plots stratified by variance bins for the MSE performance (Fig. 4f) in order to directly match the*

*Pearson correlation variance bin box plots (Fig. 4e) and make a direct comparison between Pearson correlation and MSE. We have additionally changed the supplementary figures regarding probe-wise MSE to the boxplot format instead of line graph in order to match the Pearson correlation figures.*

*We increased the label and title font sizes on all the plots in Figure 4 and moved four of the subfigures to the supplement to make the main figure less crowded. We have also increased the fontsize of all probe-wise performance supplementary figures.*

*Below is the updated Figure 4 with only the new figure legend text provided.*

***“Figure 4. Probe-wise performance of imputed species-tissue combination mean samples. e)*** *Boxplot of the probe-wise Pearson correlation with held-out observed values for the subset of highest-coverage probes in each mean inter-tissue variance quartile. Each variance quartile represented in the boxplots correspond to the variance quartile in the 2-d histograms from c. Corresponding plot when considering all probes can be found in Supplementary Fig. 12b.* ***f)*** *Boxplot of the probe-wise MSE for each mean inter-tissue variance quartile. Same format as e. Corresponding plot when considering all probes can be found in Supplementary Fig. 14b.”*

2)	The full set of imputed species-tissue combination mean samples is available only on a

UCLA web server. Could you deposit this in a permanent repository such as GEO or Zenodo

*Reply: Thank you for the suggestion. We have added the full imputed dataset to Zenodo at*

*10.5281/zenodo.13376704.*

# *References*

1. *Zou LS, Erdos MR, Taylor DL, Chines PS, Varshney A, Parker SCJ, et al. BoostMe accurately predicts DNA methylation values in whole-genome bisulfite sequencing of multiple human tissues. BMC Genomics. 2018 May 23;19(1):390.*
2. *Yu F, Xu C, Deng HW, Shen H. A novel computational strategy for DNA methylation imputation using mixture regression model (MRM). BMC Bioinformatics. 2020 Dec 1;21(1):552.*
3. *Angermueller C, Lee HJ, Reik W, Stegle O. DeepCpG: accurate prediction of single-cell DNA methylation states using deep learning. Genome Biol. 2017 Apr 11;18(1):67.*
4. *Tian Q, Zou J, Tang J, Fang Y, Yu Z, Fan S. MRCNN: a deep learning model for regression of genome-wide DNA methylation. BMC Genomics. 2019 Apr 4;20(2):192.*
5. *Qiu YL, Zheng H, Gevaert O. Genomic data imputation with variational auto-encoders. GigaScience. 2020 Aug 1;9(8):giaa082.*
6. *Ernst J, Kellis M. Large-scale imputation of epigenomic datasets for systematic annotation of diverse human tissues. Nat Biotechnol. 2015 Apr;33(4):364–76.*
7. *Durham TJ, Libbrecht MW, Howbert JJ, Bilmes J, Noble WS. PREDICTD PaRallel Epigenomics Data Imputation with Cloud-based Tensor Decomposition. Nat Commun. 2018 Apr 11;9(1):1402.*
8. *Schreiber J, Durham T, Bilmes J, Noble WS. Avocado: a multi-scale deep tensor factorization method learns a latent representation of the human epigenome. Genome Biol. 2020 Mar 30;21(1):81.*
9. *Schreiber J, Hegde D, Noble W. Zero-shot imputations across species are enabled through joint modeling of human and mouse epigenomics. In: Proceedings of the 11th ACM*

*International Conference on Bioinformatics, Computational Biology and Health Informatics*

*[Internet]. New York, NY, USA: Association for Computing Machinery; 2020 [cited 2024 Jun*

*20]. p. 1–9. (BCB ’20). Available from: https://dl.acm.org/doi/10.1145/3388440.3412412*

10. *Choi J, Chae H. methCancer-gen: a DNA methylome dataset generator for user-specified cancer type based on conditional variational autoencoder. BMC Bioinformatics. 2020 May 11;21(1):181.*
11. *Intra-Frame Coding Using a Conditional Autoencoder | IEEE Journals & Magazine | IEEE Xplore [Internet]. [cited 2024 Jun 18]. Available from:*

*https://ieeexplore.ieee.org/abstract/document/9244548*

12. *Oza P, Patel VM. C2AE: Class Conditioned Auto-Encoder for Open-Set Recognition. In 2019 [cited 2024 Jun 18]. p. 2307–16. Available from: https://openaccess.thecvf.com/content_CVPR_2019/html/Oza_C2AE_Class_Conditioned_Au to-Encoder_for_Open-Set_Recognition_CVPR_2019_paper.html*
13. *Choi Y, El-Khamy M, Lee J. Variable Rate Deep Image Compression With a Conditional Autoencoder. In 2019 [cited 2024 Jun 18]. p. 3146–54. Available from: https://openaccess.thecvf.com/content_ICCV_2019/html/Choi_Variable_Rate_Deep_Image_ Compression_With_a_Conditional_Autoencoder_ICCV_2019_paper.html*
14. *Lim J, Ryu S, Kim JW, Kim WY. Molecular generative model based on conditional variational autoencoder for de novo molecular design. J Cheminformatics. 2018 Jul 11;10(1):31.*
15. *Harvey W, Naderiparizi S, Wood F. Conditional Image Generation by Conditioning Variational Auto-Encoders [Internet]. arXiv; 2022 [cited 2024 Jun 18]. Available from: http://arxiv.org/abs/2102.12037*
16. *Fang L, Zeng T, Liu C, Bo L, Dong W, Chen C. Transformer-based Conditional*

*Variational Autoencoder for Controllable Story Generation [Internet]. arXiv; 2021 [cited 2024*

*Jun 18]. Available from: http://arxiv.org/abs/2101.00828*

17. *Kendall A, Gal Y. What Uncertainties Do We Need in Bayesian Deep Learning for Computer Vision? In: Advances in Neural Information Processing Systems [Internet]. Curran Associates, Inc.; 2017 [cited 2024 Jun 18]. Available from:*

*https://papers.nips.cc/paper_files/paper/2017/hash/2650d6089a6d640c5e85b2b88265dc2bAbstract.html*

18. *Arneson A, Haghani A, Thompson MJ, Pellegrini M, Kwon SB, Vu H, et al. A mammalian methylation array for profiling methylation levels at conserved sequences. Nat Commun. 2022 Feb 10;13(1):783.*
19. *Zhang J, Dai Y, Xiang M, Fan DP, Moghadam P, He M, et al. Dense Uncertainty Estimation [Internet]. arXiv; 2021 [cited 2024 Jun 17]. Available from:*

*http://arxiv.org/abs/2110.06427*

20. *Computation | Free Full-Text | Conditional Variational Autoencoder for Learned Image Reconstruction [Internet]. [cited 2024 Jun 18]. Available from: https://www.mdpi.com/2079-3197/9/11/114*
21. *Ran X, Xu M, Mei L, Xu Q, Liu Q. Detecting out-of-distribution samples via variational auto-encoder with reliable uncertainty estimation. Neural Netw. 2022 Jan 1;145:199–208.*
22. *Exploiting Probabilistic Siamese Visual Tracking with a Conditional Variational Autoencoder | IEEE Conference Publication | IEEE Xplore [Internet]. [cited 2024 Jun 17].*

*Available from: https://ieeexplore.ieee.org/abstract/document/9561757*

23. *Semi-conditional variational auto-encoder for flow reconstruction and uncertainty quantification from limited observations | Physics of Fluids | AIP Publishing [Internet]. [cited*

*2024 Jun 18]. Available from: https://pubs.aip.org/aip/pof/article/33/1/017119/1061258*

24. *Böhm V, Lanusse F, Seljak U. Uncertainty Quantification with Generative Models*

*[Internet]. arXiv; 2019 [cited 2024 Jun 18]. Available from:* [*http://arxiv.org/abs/1910.10046*](http://arxiv.org/abs/1910.10046)

**Second round of review**

**Reviewer 1**

The authors have addressed our comments. The revised manuscript is clear and impactful.

**Reviewer 2**

I appreciate the thoughtful and thorough responses to all my questions, including many new analyses. I think the resulting version is a high quality paper that should be published. However, I would ask the authors to consider making the Conclusion a bit more user-friendly for non-experts, who are a significant part of the intended audience of potential users. While the authors have added the term “sample-wise” in response to my original concerns regarding probe-wise correlations, I’m not sure the significance of this will be obvious to these readers. I would ask them to consider expanding on the sentence that reads “…the imputed samples maintain expected species and tissue signals”, to add something to suggest that potential users might exercise more caution if they are specifically interested in studying tissue-specific methylation at specific regions (for instance, those working on gene regulation, or its genetic/evolutionary origins). If the authors do not agree that this is helpful, I am fine with whatever decision they and the editors will best serve the readers. In any case, I commend the authors on this fine work and recommend its publication.

Ben Berman, Department of Developmental Biology and Cancer Research,
Faculty of Medicine, Hebrew University, Jerusalem, Israel

**Authors Response**

**Point-by-point responses to the reviewers’ comments:**

Reviewer #1:

The authors have addressed our comments. The revised manuscript is clear and impactful.

*Reply: We thank the reviewer for the positive comment and again for their review.*

Reviewer #2:

I appreciate the thoughtful and thorough responses to all my questions, including many new analyses. I think the resulting version is a high quality paper that should be published. However, I would ask the authors to consider making the Conclusion a bit more user-friendly for non-experts, who are a significant part of the intended audience of potential users. While the authors have added the term “sample-wise” in response to my original concerns regarding probe-wise correlations, I’m not sure the significance of this will be obvious to these readers. I would ask them to consider expanding on the sentence that reads “…the imputed samples maintain expected species and tissue signals”, to add something to suggest that potential users might exercise more caution if they are specifically interested in studying tissue-specific methylation at specific regions (for instance, those working on gene regulation, or its genetic/evolutionary origins). If the authors do not agree that this is helpful, I am fine with whatever decision they and the editors will best serve the readers. In any case, I commend the authors on this fine work and recommend its publication.

Ben Berman, Department of Developmental Biology and Cancer Research, Faculty of Medicine, Hebrew University, Jerusalem, Israel

*Reply: We thank the reviewer again for the review and noting the improvements to the manuscript. We also thank the reviewer for the additional comment which we address below.*

*We have edited the conclusion to be more user-friendly for non-experts by replacing*

*“sample-wise” and “probe-wise” correlation with “correlation across probes within a sample” and “correlation across samples within a probe”. We have also added a sentence explaining that while the probe-wise correlation outperforms baseline, we still see a decrease in performance on probes with higher tissue-to-tissue variability.*

*Modified text: “We have shown that CMImpute achieves strong correlation across probes within a sample and low MSE with observed values. While CMImpute’s correlation across samples within a probe was not as strong, CMImpute still achieves high correlation though did decrease in performance in probes that are highly variable across tissues. We have also shown*

*CMImpute’s predictions surpass several baseline methods for all measures considered.”*

*Additionally, we expanded on the sentence that reads “...the imputed samples maintain expected species and tissue signals” to clarify that the computational predictions are subject to error which could impact downstream analyses. We believe that these changes in conjunction with the changes shown above highlight CMImpute’s strengths while making potential shortcomings known.*

*Modified text: “We demonstrated that the imputed samples maintain similar species and tissue relationships as with the observed data. We expect that these imputed samples will be useful for* *downstream analyses, though we caveat the samples are computational predictions and we note the most variable probes are the hardest to accurately impute.”*
